# The role of microenvironment in tumor angiogenesis

**DOI:** 10.1186/s13046-020-01709-5

**Published:** 2020-09-30

**Authors:** Xianjie Jiang, Jie Wang, Xiangying Deng, Fang Xiong, Shanshan Zhang, Zhaojian Gong, Xiayu Li, Ke Cao, Hao Deng, Yi He, Qianjin Liao, Bo Xiang, Ming Zhou, Can Guo, Zhaoyang Zeng, Guiyuan Li, Xiaoling Li, Wei Xiong

**Affiliations:** 1 NHC Key Laboratory of Carcinogenesis, Hunan Cancer Hospital and the Affiliated Cancer Hospital of Xiangya School of Medicine, Central South University, Changsha, China; 2 The Key Laboratory of Carcinogenesis and Cancer Invasion of the Chinese Ministry of Education, Cancer Research Institute and School of Basic Medicine Sciences, Central South University, Changsha, China; 3 Department of Stomatology, Xiangya Hospital, Central South University, Changsha, China; 4 Department of Oral and Maxillofacial Surgery, The Second Xiangya Hospital, Central South University, Changsha, China; 5 Hunan Key Laboratory of Nonresolving Inflammation and Cancer, Disease Genome Research Center, The Third Xiangya Hospital, Central South University, Changsha, China

**Keywords:** Tumor angiogenesis, Tumor microenvironment, Angiogenic factor, Inflammatory factor, Hypoxia inhibitor

## Abstract

Tumor angiogenesis is necessary for the continued survival and development of tumor cells, and plays an important role in their growth, invasion, and metastasis. The tumor microenvironment—composed of tumor cells, surrounding cells, and secreted cytokines—provides a conducive environment for the growth and survival of tumors. Different components of the tumor microenvironment can regulate tumor development. In this review, we have discussed the regulatory role of the microenvironment in tumor angiogenesis. High expression of angiogenic factors and inflammatory cytokines in the tumor microenvironment, as well as hypoxia, are presumed to be the reasons for poor therapeutic efficacy of current anti-angiogenic drugs. A combination of anti-angiogenic drugs and antitumor inflammatory drugs or hypoxia inhibitors might improve the therapeutic outcome.

## Background

Nutrients, oxygen, metabolites, chemical mediators, and metabolic waste can be transported through blood vessels between cells to maintain homeostasis of the immune system, body temperature, and pH [1]. Blood vessels play an important role in embryonic development, body growth, and wound healing. Neovascularization is an important process for the growth and metastasis of tumors, and is used to transport nutrients and remove metabolic waste from tumor cells. Several studies have showed that neovascularization is essential for tumor growth beyond 1–2 mm in diameter [2, 3]. During vasculogenesis, endothelial progenitor cells derived from hemangioblasts are recruited and differentiate into mature vascular endothelial cells when stimulated by the local environment and eventually form blood vessels [4]. The physiological process through which new blood vessels are formed from pre-existing blood vessels is called angiogenesis. The angiogenic process is divided into the following steps: pro-angiogenic factors are initially secreted into the extracellular fluid to activate endothelial cells. These endothelial cells migrate along the concentration gradient of pro-angiogenic factors and attach to the blood vessels to form a functional vascular network [5]. Tumor tissues have high angiogenic capacity. Blood vessels in tumor tissues are primarily composed of endothelial cells. Blood capillaries in normal tissue undergo expansion under ischemic or hypoxic conditions, resulting in a marked increase in capillary permeability and fibrin exudation. Simultaneously, collagenase activation and basement membrane rupture can promote extracellular matrix remodeling. In addition, angiogenic factors induce endothelial cell proliferation, and new endothelial cells are assembled into tubular structures to form new tumor vessels [6, 7]. Another form of angiogenesis found in tumor tissues is vasculogenic mimicry. This is the ability of tumor cells to form tubular structures similar to those formed by endothelial cells under the influence of external stimuli. Erythrocytes are present in the lumen of these tubular structures. Moreover, these tubular tissues can attach to endothelial blood vessels to form a complete vascular network [8]. Vasculogenic mimicry can accelerate the formation of new blood vessels in tumor tissues and promote tumor growth, invasion, and metastasis. Tumor neovascularization provides nutrients and oxygen to tumor cells and removes metabolic waste. It prevents the accumulation of acidic metabolites and facilitates the growth of tumor cells. In addition, tumor neovascularization can also affect the microenvironment of the tumor. Tumor cells can metastasize from their primary location along the walls of new blood vessels throughout the body and begin to grow to form new tumors in the right places [9]. Tumor neovascularization can cause tumor immunosuppression by inhibiting dendritic cell (DC) maturation and antigen presentation, recruitment of immunosuppressive cells, and inhibiting cytotoxic T cell activity through angiogenic factors [10]. In addition, tumor neovascularization is immature and the lack of mural cell adhesion leads to tumor vascular hyperpermeability, poor perfusion, and hypoxia without much improvement. Increased hypoxia in solid tumors further accelerates tumor growth and metastasis [11, 12]. The tumor microenvironment, in turn, produces a large number of factors that promote tumor angiogenesis, forming a malignant tumor growth-promoting cycle [13].

### Hypoxia and its evolutionary role during angiogenesis

**Fig. 1:**
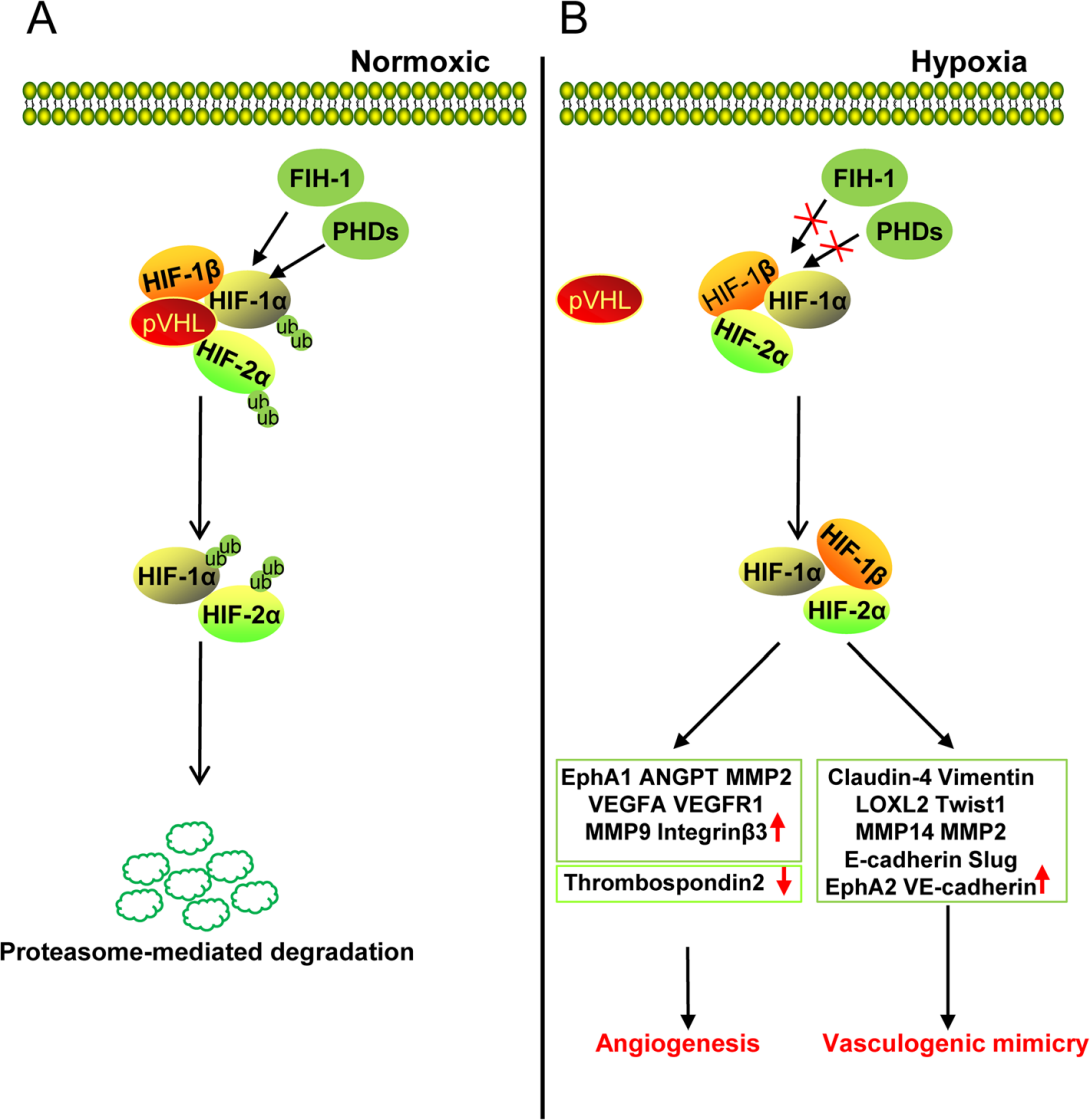
The role of hypoxia in tumor angiogenesis**. a **Under normoxic conditions, HIF-1α and HIF-2α are hydroxylated by PHDs and FIH-1. Subsequently, pVHL can recognize and ubiquitinate hydroxylated HIF-1α/HIF-2α and degrade them via proteasome-mediated degradation. **b** Under hypoxic conditions, the inactivation of FIH-1 and PHDs cannot hydroxylate HIF-1/HIF-2α, decreases HIFα-VHL binding, and promotes the formation of HIFα-HIFβ dimers that enter the nucleus to activate downstream targets. HIF-1α/HIF-2α can activate EphA1, ANGPT, VEGFA, VEGFR1, and other angiogenesis related genes to promote tumor angiogenesis. Alternatively, HIF-1α/HIF-2α can activate Claudin-4, Vimentin, LOXL2, Twist1, VE-cadherin to promote vasculogenic mimicry

During the development of solid tumors, a large amount of nutrients is consumed due to rapid proliferation of tumor cells. Moreover, high oxygen consumption, lack of nutrients, and accumulation of metabolic substances in cells can create an oxygen-deficient microenvironment that is not suitable for tumor cell growth [14]. However, tumor cells can undergo metabolic reprogramming by changing the expression of glycolysis-related proteins, such as GLUT1, GLUT3, LDHA, and PKM2 under hypoxic conditions and increase glucose uptake to promote their growth [15]. Furthermore, the hypoxic microenvironment can induce tumor cells to alter the expression of epithelial-mesenchymal transition (EMT) markers such as N-cadherin, E-cadherin, slug, snail, and vimentin, and increase the production of matrix metalloproteinases (MMPs) that promote invasive metastasis [16, 17]. Hypoxia-inducible factor (HIF) is highly expressed in the hypoxic tumor microenvironment. HIF is a dimeric transcription factor composed of HIF-1α or HIF-2α and HIF-1β/ARNT subunits. Under normoxic conditions, the HIFα protein is hydroxylated in the presence of proline hydroxylase (PHD) and aspartate hydroxylase (factor-inhibiting HIF (FIH)). The hydroxylated HIFα subunit binds to the E3 ubiquitinated ligase Hippel-Lindau (VHL) protein. Subsequently, HIFα is recognized and ubiquitinated by the ubiquitin ligase system, resulting in proteasomal degradation of HIFα protein. The hydroxylation status of proline residues in HIFα is the key factor for VHL binding. PHD inactivation under hypoxic conditions decreases HIFα-VHL binding and promotes the formation of HIFα-HIFβ dimers that enter the nucleus to activate E-box-like hypoxic response elements (HREs) on the promoter of downstream targets [18]. Recent studies have shown that hypoxia plays an important role in promoting tumor angiogenesis (Fig. 1). HIF-1α can transcriptionally activate several pro-angiogenesis molecules by directly binding to their promoters. HIF-1α can bind to vascular endothelial growth factor (VEGF) and VEGF receptor 1 (VEGFR1) gene promoter at the HRE site, and induce the transcription of *VEGFA* and *VEGFR1* genes [19]. HIF-1α-induced *VEGF* and *ANGPTL4* expression can effectively promote tumor angiogenesis in melanoma. However, downregulation of *VEGF* or *ANGPTL4* expression can block this process [20]. In hepatocellular carcinoma tumors, HIF-1α promotes angiogenesis through transcriptional activation of downstream target genes including *VEGFA*, *VEGFR1*, and *EphA1*. Inhibition of HIF-1α-binding protein CDK5 can suppress the transcriptional activity of HIF-1α, leading to downregulation of HIF-1α downstream angiogenic target genes and inhibition of angiogenesis in hepatocellular carcinoma [21]. Furthermore, increased VEGFR2 expression under hypoxic conditions can promote angiogenesis. Instead of activating VEGFR2 via HIF-induced transcription, hypoxia increases phosducin-like 3 (PDCL3) production to stabilize VEGFR2 protein expression [22]. In addition, HIF-1α can reduce the expression of anti-angiogenic molecules. Moreover, thrombospondin 2 mRNA expression can be decreased under hypoxic conditions by targeting HIF-1α. These results suggest that HIF-1α can promote tumor angiogenesis not only by activating pro-angiogenic genes, but also inhibiting anti-angiogenic genes under hypoxic conditions [23]. Hypoxia can also regulate the expression of various components of the extracellular matrix (ECM) to promote tumor angiogenesis. Hypoxia has been shown to induce the expression of MMP2 and MMP9, which are important molecules for tumor cell invasion and metastasis [23–25]. In addition, hypoxia-induced integrin β3 expression can affect endothelial cell tube formation [26].

Hypoxia also plays an important role in promoting vasculogenic mimicry in various tumors. In colorectal cancer, hypoxic microenvironment-induced HIF-1α expression upregulates EMT-related molecules such as claudin-4, vimentin, and E-cadherin, promoting EMT-induced vasculogenic mimicry [27]. In ovarian cancer, hypoxia can promote EMT-induced vasculogenic mimicry by upregulating E-cadherin, Twist1, Slug, and VE-cadherin [28]. In liver cancer, EMT-induced vasculogenic mimicry is achieved by increased expression of HIF-1α-induced LOXL2 [29]. VE-cadherin can also regulate vasculogenic mimicry by phosphorylating and activating EphA2; activated EphA2 can phosphorylate FAK to reactivate the extracellular signal-regulated kinase ERK1/2. In addition, EphA2 and VE-cadherin can activate PI3K signaling and MMP14/MMP2, and promote the cleavage of laminin5γ2 into 5γ2 and 5γ2x fragments. Increased levels of these fragments in the extracellular microenvironment can eventually form vasculogenic mimicry network structures [30]. In glioma, both HIF-1α and HIF-2α bind directly to the VE-cadherin promoter and increase VE-cadherin expression to promote vasculogenic mimicry [31]. A similar phenomenon was demonstrated in esophageal cancer [32]. In melanoma, hypoxia-induced VE-cadherin expression is regulated by Bcl-2. Short-interfering RNA (siRNA)-mediated silencing of Bcl-2 expression can markedly inhibit vasculogenic mimicry in melanoma under hypoxic conditions [33]. In pancreatic cancer, HIF-2α induces VE-cadherin expression to promote vasculogenic mimicry by upregulating Twist1 expression. The binding of Twist1 to the VE-cadherin promoter increases VE-cadherin expression, which consequently, promotes the formation of vasculogenic mimicry [34]. These results indicate that hypoxia-inducible factors can regulate VE-cadherin expression using diverse mechanisms in different tumors. In nasopharyngeal carcinoma, EBV-induced angiogenesis mimicry is primarily achieved through the PI3K/AKT/mTOR/HIF-1α/VEGFA signaling cascade. Moreover, HIF-1α and VEGF inhibitors can effectively inhibit vasculogenic mimicry in nasopharyngeal carcinoma. Therefore, HIF-1α plays an important role in vasculogenic mimicry of nasopharyngeal carcinoma [35]. HIF-1α/NRP-1 in fibrosarcoma and HIF-1α in cholangiocarcinoma can promote vasculogenic mimicry under hypoxic conditions [36]. In conclusion, HIF-1α and vasculogenic mimicry can be used as independent prognostic factors for the overall survival of patients. In addition to the hypoxic microenvironment, there are several factors in the tumor microenvironment that can promote tumor angiogenesis.

### Tumor microenvironment and its evolutionary role during angiogenesis

**Fig. 2:**
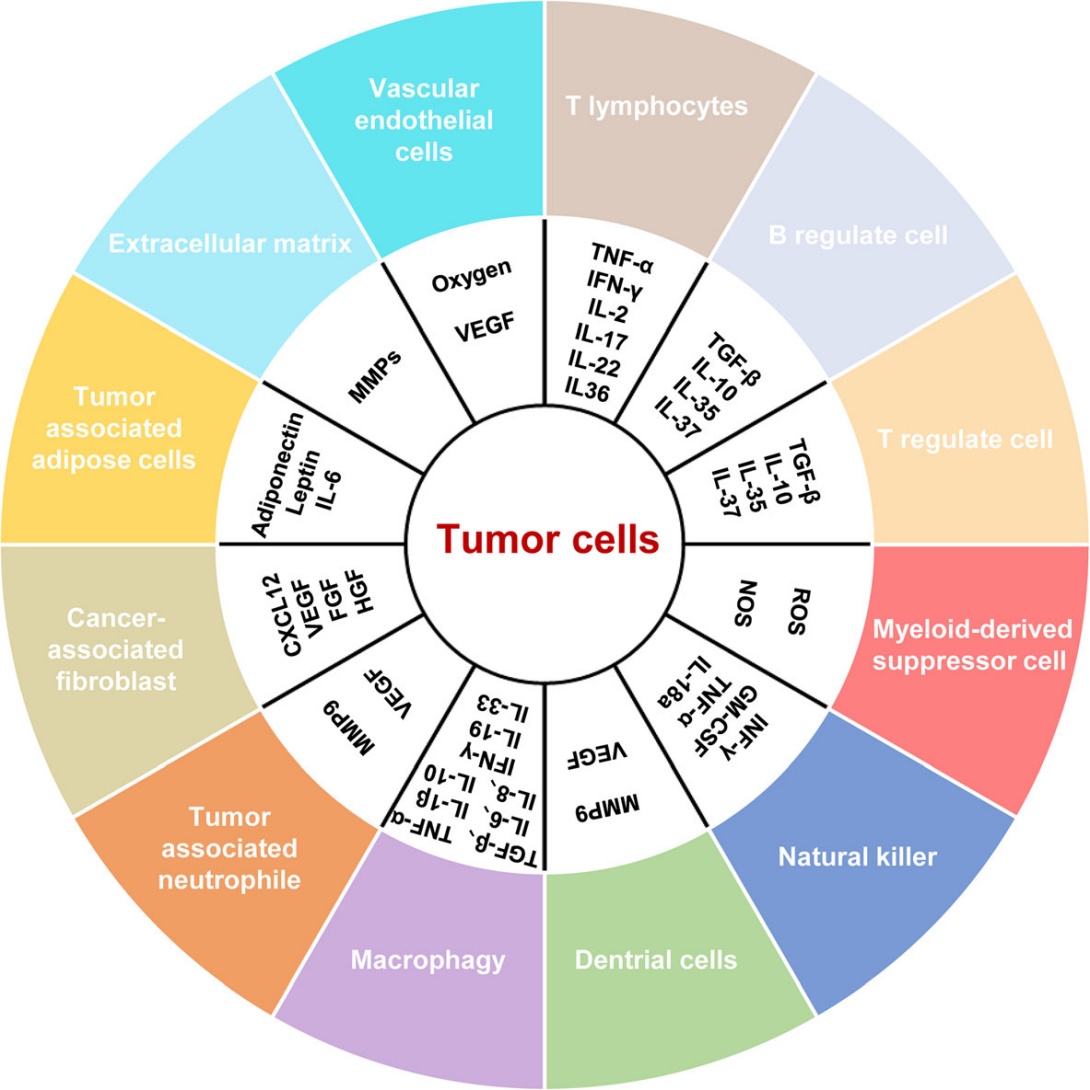
Cytokines and cell growth factors secreted in the tumor microenvironment

Malignant tumor cells recruit normal cells around tumor tissue to form a complex structure consisting of both malignant and non-transformed cells [37]. This environment composed of tumor cells, endothelial cells, immune cells, fibroblasts, macrophages, and the extracellular matrix surrounding or infiltrating tumor tissues, as well as soluble substances such as cytokines and growth factors secreted by these cells is called the tumor microenvironment [38] (Fig. 2). Immune cells play an important role in providing protection against invading foreign pathogens and eliminating damaged cells and tumor cells from the body. There are two types of tumor-infiltrating T cells: CD4^+^ T cells (helper T cells) and CD8^+^ T cells (cytotoxic T cells). T cells are the most important effector cells of the human immune system; these cells exert their antitumor effect by secreting cytokines such as tumor necrosis factor-alpha (TNF-α), interferon gamma (IFN-γ), interleukin (IL)-2, IL-17, IL-22, and IL-36 [39, 40]. Regulatory B cells and regulatory T cells are the major immunosuppressive cells of the immune system. These cells secrete transforming growth factor beta (TGF-β), IL-10, IL-35, IL-37, and other cytokines to suppress the immune response of lymphocytes. Myeloid-derived suppressor cells can increase reactive oxygen species and nitric oxide synthase production to suppress the immune response of cytotoxic T cells. Moreover, these immunosuppressive cells can prevent overactivation of the immune system and maintain immune system homeostasis [41–43]. Peripheral lymphocytes consist of approximately 10% of natural killer (NK) cells. These cells are also found in the spleen, peripheral blood, bone marrow, and lymph nodes. They migrate to the inflammatory sites driven by chemokines. Activated NK cells secrete large amounts of IFN-γ, granulocyte-macrophage colony-stimulating factor, TNF-α, IL-18, and other factors to inhibit tumor growth [44, 45]. DCs secrete inflammatory cytokines, promote Th1 cell activation, and induce a cytotoxic response [46]. Tumor-infiltrating neutrophils secrete large amounts of MMPs and growth factors, such as MMP9 and VEGF, to promote proliferation, invasion, and metastasis of tumor cells [47]. Macrophages are cells of the innate immune system that play an important role in tissue homeostasis. They can engulf and digest cellular debris, and activate immune cells to respond to and eliminate pathogens. In tumor tissues, macrophages are classified into M1 and M2 types. M1 macrophages are conventionally activated macrophages that secrete pro-inflammatory cytokines such as IL-1β, IL-6, IL-8, IL-18, TNF-α, and IFN-γ, and exert an anti-tumor effect. M2 macrophages are alternatively activated macrophages that secrete IL-4, IL-10, IL-19, IL-33, TGF-β, and epithelial growth factor, all of which play an important role in promoting tumor growth and metastasis [48–51].

Mesenchymal cells and fibroblasts are also present in large numbers in tumor tissues. These cells secrete growth factors such as fibroblast growth factor (FGF), VEGF, MMP2, and CXCL-12 chemokine to promote growth, invasion, and metastasis of tumor cells[52, 53]. Adipose tissue secretes IL-6, adiponectin, and leptin to promote malignant tumor growth [54–56]. Vascular endothelial cells provide oxygen to the tumor microenvironment by forming new blood vessels. In addition, VEGF secretion can promote endothelial cell proliferation and tubule formation. The ECM is composed of intercellular substances and the basement membrane, and contains tumor cells, cytokines, growth factors, and various MMPs secreted by tumor cells or other cells in the tumor microenvironment. In addition, acidic substances in tumor cell metabolites maintain the acidic microenvironment in tumor tissues, which in turn promotes epithelial-mesenchymal transition (EMT) of tumor cells. The rapid growth of tumor cells requires enormous energy. Moreover, high consumption of energy increases oxidative phosphorylation capacity to fulfill the growth demand of the cell. However, the rate of vascular regeneration in tumor tissues is often difficult to match with the growth rate of tumor cells. Therefore, the tumor microenvironment is typically hypoxic. Recent studies have demonstrated that high expression of non-coding RNA in the microenvironment plays an important role in tumor growth and migration [57].

Increased angiogenesis in tumor tissues can increase the supply of nutrients to tumor cells, and facilitate tumor growth, invasion, and metastasis. Recent studies have showed that several cytokines in the tumor microenvironment and some conventional anticancer agents exhibit a pro-angiogenic effect. Herein, we reviewed the role of the microenvironment in tumor angiogenesis. A list of current Food and Drug Administration (FDA)-approved drugs for tumor angiogenesis has also been provided (Table 1). We believe that a combination of anti-angiogenic inhibitors and anti-inflammatory drugs, or hypoxia inhibitors can improve the therapeutic outcome.

**Table 1:** Summary of FDA-approved anti-angiogenic agents

Anti-angiogenic agents	Manufacturer	Target	Date of first FDA approval	Condition
Bevacizumab (Avastin)	Genentech	VEGF	2004. 02	Metastatic colorectal cancer, non-squamous small cell lung cancer, cervical cancer, ovarian cancer, metastatic breast cancer, malignant glioma
Ramucirumab (Cyramza)	ImClone	VEGFR2	2014. 04	Advanced gastric or gastroesophageal adenocarcinoma, non-small-cell lung cancer, and metastatic urinary tract epithelial cancer
Ziv-aflibercept (Zaltrap)	Sanofi	VEGFA/VEGFB /PIGF	2012. 08	Metastatic colorectal cancer
Axitinib (Inlyta)	Pfizer	VEGFR/KIT/PDGFR/RET/ CSF1R/FLT3	2012. 01	Advanced renal cell carcinoma
Sorafenib (Nexavar)	Bayer	VEGFR2/PDGFR /KIT/FLT3/BRAF	2005. 12	Renal cell and hepatocellular carcinoma and thyroid cancer
Sunitinib (Sutent)	Pfizer	VEGFR/KIT /PDGFR	2006. 01	Gastrointestinal stromal tumors, advanced renal cancer, and metastatic well-differentiated advanced pancreatic neuroendocrine tumors
Regorafenib (Stivqrga)	Bayer	VEGFR/PDGFR /KIT/FGFR	2012. 09	Metastatic colorectal cancer, gastrointestinal mesenchymal liver cancer
Nintedanib (OFEV)	Boehringer lngelheim	VEGFR/PDGFR /FGFR	2014. 10	Idiopathic pulmonary fibrosis, non-small cell lung cancer
Cabozantinib (Cabometyx)	Exelixis	RET/VEGFR2 /PDGFR/KIT/FLT3/ MET/AXL	2012. 11	Metastatic thyroid cancer, non-small cell lung cancer with c-Met amplification
Pazopanib (Votrient)	GlaxoSmithKline	VEGFR/PDGFR /KIT	2009. 10	Advanced renal cancer, advanced soft tissue sarcoma, epithelial ovarian cancer, non-small cell lung cancer

### Regulation of angiogenesis in the tumor microenvironment

Tumor angiogenesis is an important process by which tumor cells can grow, invade, and metastasize. Tumor angiogenesis is positively correlated with tumor malignancy. Angiogenic factors, cytokines, and free non-coding RNAs in the tumor microenvironment can promote tumor angiogenesis. The regulatory mechanisms of tumor angiogenesis in the presence of angiogenic factors, cytokines, and non-coding RNAs in the tumor microenvironment are described below.

#### Angiogenic factors

A wide variety of protein polypeptides are distributed in an organism. Some of these protein polypeptide factors have a role in promoting neovascularization and are known as angiogenic factors. These play an important role in regulating both normal and abnormal angiogenesis. The most important of these for tumor angiogenesis are the three peptide families of VEGF, FGF, and platelet-derived growth factor (PDGF).

#### VEGF plays a pivotal role in tumor angiogenesis

**Fig. 3:**
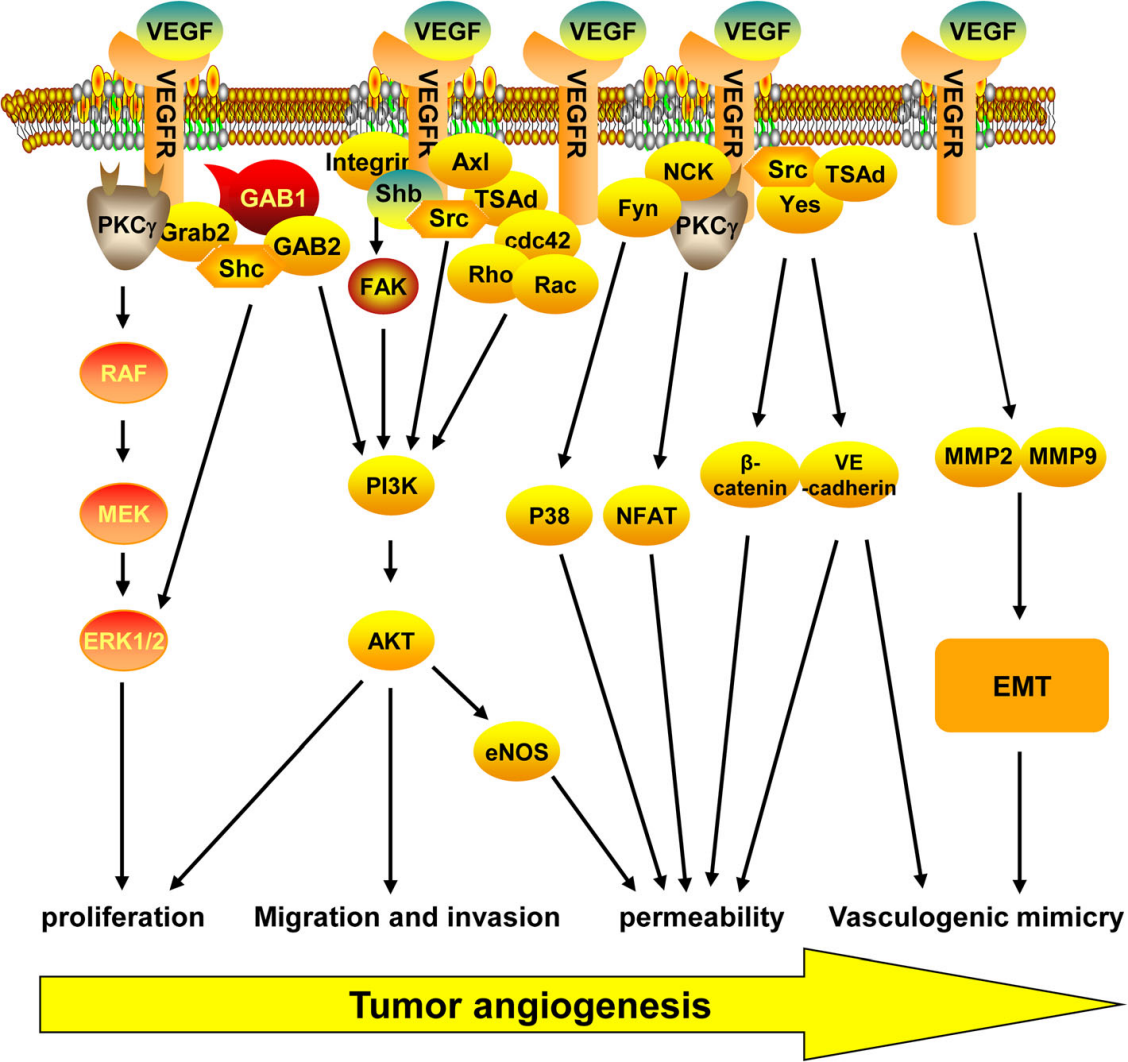
Schematic representation of key VEGF/VEGFR signal transduction pathways**.** **Proliferation:** VEGFR can interact with Grab/Src/Gab1/Shb/PKCγ to activate RAF/MEK/MAPK and PI3K/AKT signaling pathways, and promote the proliferation of endothelial cells. **Migration and invasion: **VEGFR can activate PI3K/AKT by binding to cdc42, Rho, and RacGTPases, and promotes the migration and invasion of endothelial cells. **Permeability**: VEGFR can enhance blood vessel permeability by activating NFAT/β-catenin/VE-cadherin, and eNOS. **Vasculogenic mimicry: **VEGFR can promote EMT-induced vasculogenic mimicry by upregulating the expression of EMT-related genes

VEGF is a 40–45 kD dimeric cysteine-rich protein that was discovered in 1983 and is highly conserved among mammals. It was found to increase the permeability of tumor blood vessels and promote the formation of ascites [58]. In 1989, the VEGF protein was first isolated and its role in the process of angiogenesis was identified [59, 60]. The human VEGF family has multiple members. Among them, VEGFA was identified first, and is the most specific angiogenesis-inducing factor. VEGF is commonly referred to as VEGFA. The *VEGFA* gene located on chromosome 6p21. 3 extends over 28 kb in length and consists of eight exons and seven introns. *VEGFA* mRNA undergoes alternative splicing during its maturation and generates seven spliceosomes: VEGF121, VEGF145, VEGFA162, VEGF165, VEGF183, VEGF189, and VEGF206. Each spliceosome can bind to different receptors and perform different functions [61]. VEGF regulates tumor angiogenesis by binding to its receptor (VEGFR1–3) and activates intracellular signaling pathways (Fig. 3). The VEGF receptor can be divided into three domains: extracellular VEGF-binding domain, transmembrane domain, and intracellular domain (tyrosine activation domain). VEGF binds to the extracellular domain of VEGFR to phosphorylate intracellular tyrosine residues of VEGFR, and activates intracellular signaling pathways [62]. VEGFR1 and VEGFR2 are predominantly expressed in vascular endothelial cells; however, high expression is also observed in macrophages and tumor cells [63]. VEGFR3 is highly expressed in endothelial lymphocytes [64]. It is well known that endothelial cells play a pivotal role in tumor angiogenesis; therefore, the regulation of endothelial cell signaling plays an important role in tumor angiogenesis and development. Studies have shown that the binding of VEGF to VEGFR2 can induce VEGFR2 phosphorylation at Y1175, leading to PKCγ activation, Raf-MEK-MAPK signal transduction, and proliferation of endothelial cells [65, 66]. Moreover, VEGF has also been reported to promote endothelial cell proliferation by activating MAPK signaling via growth factor receptor binding 2 and Shc [67]. In addition, VEGF-activated VEGFR2 can activate PI3K/Akt signaling via direct binding with Gab1/Gab2, and promote the proliferation of vascular endothelial cells [68]. VEGF can also modulate FAK activity by VEGFR2 activation of c-Src, shb, or RhoA, or binding with integrin proteins to alter PI3K/Akt signaling and endothelial cell proliferation [69–72]. Furthermore, Axl plays an important role in VEGF-mediated Src/TSAd activation of PI3K/Akt [73]. The migration of endothelial cells is an important prerequisite for tumor angiogenesis. PI3K/Akt signaling is responsible for the expression of MMPs and other molecules required for invasion and metastasis of tumor cells [74]. Additionally, PI3K/Akt signaling can activate Cdc42, Rho, and Rac proteins, and promote VEGF-mediated invasion and metastasis of endothelial cells [75, 76]. Vascular permeability is required for tumor angiogenesis; Src plays an important role in VEGF-induced vascular permeability. VEGFA can activate c-Src and Yes proteins via VEGFR and phosphorylate adhesion factors such as VE-cadherin and beta-catenin in the presence of TSAd to increase vascular permeability. In addition, the phosphorylation of VE-cadherin via VEGF-induced activation of Rac can disrupt endothelial cell-cell interaction and increase the permeability of blood vessels [77]. Furthermore, activated endothelial nitric oxide synthase (eNOS) plays an important role in vascular permeability by releasing nitric oxide in blood vessels. VEGF can activate nuclear factor of activated T-cells by activating PLCγ via the PI3K/Akt signaling pathway to modulate intracellular calcium ion concentration or increase eNOS production to increase vascular permeability [78, 79]. VEGFR can also activate the P38/MAPK signaling pathway through Nck and Fyn binding, inducing alterations in the cytoskeleton and promoting tube formation in endothelial cells. In a melanoma study, VEGF was found to promote vasculogenic mimicry by activating PI3K/Akt signaling [80]. In addition, vasculogenic mimicry markers such as VE-cadherin, MMP2, and MMP9 have been shown to be modulated by VEGFA. These results suggest that VEGFA plays an important role in vasculogenic mimicry in tumor cells. The tumor microenvironment plays a key role in tumor angiogenesis as numerous cells here can secrete VEGF protein,

#### FGF in the tumor microenvironment aids tumor angiogenesis

FGF and its receptor play an important role in cell proliferation, migration, survival, and differentiation. FGF interacts with its cofactor heparan sulfate or Klotho, and dimerizes with FGFR to exert its physiological function [81, 82]. The FGF family is divided into six subfamilies according to their sequence homology and development characteristics and are composed of 18 members in mammals. bFGF—also called FGF2—was discovered first, and plays a crucial role in tumor angiogenesis [83]. The binding of FGF to FGFR promotes autophosphorylation of FGFR, which induces a conformational change from inactive to active. Activated FGFR further activates FGFR substrate 2 and recruits PLCγ, which consequently, recruits growth factor receptor binding 2 to activate PKC, RAS/RAF/MEK/MAPK signaling and PI3K/AKT signaling. FGFR also activates the p38 MAPK and JNK signaling pathways, STAT signaling pathway, and ribosomal protein S6 kinase 2 [84, 85]. Furthermore, FGF2 activates intracellular signaling and promotes angiogenesis by interacting with the membrane-bound integrinαVβ3 [86]. FGF can modulate these signaling pathways to stimulate neovascular formation and maturation by promoting endothelial cell proliferation and ECM degradation, and altering the expression of intercellular adhesion molecules [87]. FGF2 plays an important role in tumor angiogenesis. Studies have shown that FGF2 secretion by neutrophils in the tumor microenvironment can promote angiogenesis in metastatic liver tumors [88]. Similarly, a long non-coding RNA (lncRNA), MALAT1, was found to promote angiogenesis in thyroid cancer tissues by increasing FGF2 secretion from tumor-associated macrophages [89]. Finally, FGF2 exerts a synergistic effect with PDGF-BB to increase the interaction between endothelial and mural cells, and promote tumor angiogenesis and metastasis [90]. Therefore, decreasing FGF expression in the tumor microenvironment can be an important antitumor therapeutic strategy in future.

#### Aberrant expression of PDGF promote tumor angiogenesis

PDGF plays an important role in embryonic development, cell growth and differentiation, and tissue repair. Several pathological conditions occur due to aberrant expression of PDGF and its receptors [91]. PDGFA expression is upregulated in several cancers. PDGFA increases tumor angiogenesis in both ovarian and hepatocellular carcinoma cells by promoting MEK/ERK signaling [92, 93]. PDGF-BB can induce proliferation, migration, and tube formation of vascular endothelial cells in addition to increasing VEGF expression [94, 95]. PDGF-BB can also facilitate peripheral migration of pericytes to surrounding tumors to promote tumor angiogenesis and vasculogenic mimicry formation [96, 97]. PDGF-D can promote tumor angiogenesis of colorectal cancer by activating Notch1/Twist1 signaling and recruiting macrophages to tumor tissues [98, 99].

### Cytokines

**Fig. 4:**
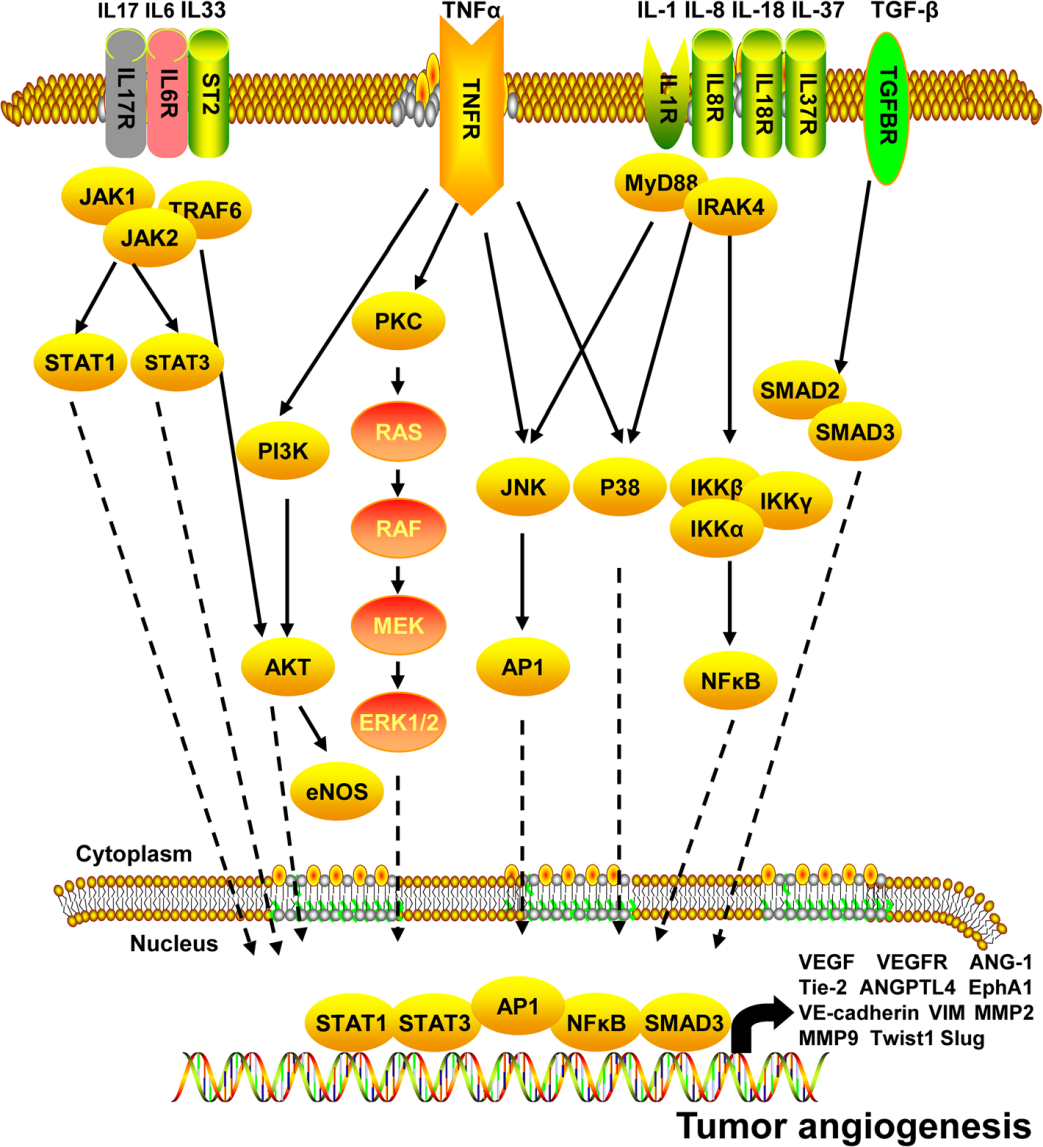
The regulatory network of tumor angiogenesis in the tumor microenvironment

Autocrine and paracrine cytokines are secreted by tumor cells in the tumor microenvironment. These cytokines play an important role in tumor growth, invasion, and metastasis. Recent studies have showed that several cytokines in the tumor microenvironment play an important role in tumor angiogenesis. The effects of cytokines on angiogenesis in the tumor microenvironment are described in Fig. 4.

#### TGF-β: a controversial pro-angiogenic cytokine

The TGF-β family of peptide signaling molecules include TGFB1-B3, activins, inhibins, Nodal, bone morphogenetic proteins (BMPs), and growth differentiation factors [100]. The TGF-β family plays an important role in embryonic development and regulation of tissue homeostasis, and its aberrant expression is associated with several diseases. TGF-β plays an important role in the growth, invasion, metastasis, and immune escape of tumors. Although, the role of TGF-β in tumor angiogenesis remains controversial. While some studies have shown that TGF-β can promote tumor angiogenesis, a few other studies have revealed its inhibitory effect. TGF-β is highly expressed in several cancers; however, high TGF-β expression is negatively correlated with patient prognosis and positively correlated with tumor growth and angiogenesis [101]. In colorectal and renal cancer cells, TGF-β overexpression promotes tumor angiogenesis, and the addition of neutralizing antibodies to TGF-β1 markedly reduces tumor angiogenesis [100]. One study demonstrated that VEGF and TGFBR1 (ALK5) inhibitors can synergistically promote tumor angiogenesis by potentially blocking the downstream effectors of ALK5 such as Smad2 and Smad3 [102]. However, according to recent studies, Smad3—a tumor-promoting factor—can increase VEGF expression and promote tumor angiogenesis, and Smad2—a tumor-suppressing factor—can inhibit tumor metastasis and angiogenesis [103]. These studies also confirmed that TGF-β inhibits tumor growth in the early stages of tumorigenesis and promotes tumor growth in the advanced stages. Therefore, the potential targeting of TGF-β for tumor therapy requires further research. BMPs can also increase tumor angiogenesis. A Matrigel plug experiment revealed that BMP2 can increase angiogenesis in lung cancer cells. Moreover, BMP2 antagonists blocked the angiogenic effect of BMP2 [104, 105]. Similar results were obtained in breast cancer and melanoma cells [106, 107]. These results suggest that BMPs can either directly induce VEGF expression or recruit endothelial precursor cells to facilitate secretion of VEGF and placental growth factor (PIGF) from mesenchymal stem cells to promote tumor angiogenesis. The regulatory mechanisms of TGF-β activity in tumor angiogenesis is not well understood and requires further research.

#### IFNs are multifaceted in the regulation of tumor angiogenesis

Interferons (IFNs) are biologically active glycoproteins secreted by cells, following bacterial or viral infection. IFNs possess antiviral, antibacterial, antitumor, and immune regulatory activity, and can inhibit angiogenesis [108]. In neuroendocrine tumors, IFN-α downregulates VEGF expression by inhibiting SP1 or SP3 and reduces angiogenesis [109]. IFN-2α downregulates HIF-1α expression by inhibiting PI3K or MAPK signaling, resulting in suppression of VEGF expression and tumor angiogenesis [110]. However, some studies have showed that IFN-α can promote the formation of vasculogenic mimicry. IFN-α can increase HIF-1a expression and promote vasculogenic mimicry in the kidney, breast, ovarian, and colorectal cancer cells by activating PI3K/AKT/mTOR signaling [111]. IFN-β can effectively inhibit endothelial precursor cell-mediated tumor angiogenesis. The inhibitory effect of INF-γ on tumor angiogenesis has been extensively reported. However, recent studies have showed that IFN-γ can promote tumor angiogenesis in mesenchymal stem cells. Moreover, IFN-γ increases HIF-1α expression in MSCs, which in turn, upregulates VEGF expression and promotes tumor angiogenesis [112].

#### TNF-α: an anti-angiogenic and pro-angiogenic factor

TNF was originally named owing to its ability to directly cause hemorrhagic necrosis in tumors. However, later studies found that in addition to killing tumor cells, TNF can function as an inflammatory mediator. TNF-α is produced by kinase-activated macrophages and bind to specific homotrimeric receptors on the cell membrane. TNF-α can activate caspase protease, JNK, and NF-κB signaling pathways to induce inflammation and promote cell growth, differentiation, and apoptosis. Previous studies have showed that TNF-α can inhibit tumor angiogenesis. However, recent studies have demonstrated that TNF-α exerts pro-angiogenic activity in tumors. TNF-α promotes human umbilical vein endothelial cell (HUVEC) migration and tube formation capacity by activating PI3K, p38, JNK, ERK, and NF-κb signaling pathways [113]. In prostate cancer cells, TNF-α induces VEGFA expression by activating downstream NF-κb signaling, and promotes endothelial cell angiogenesis. In contrast, miR-130b suppresses angiogenesis in prostate cancer cells by inhibiting TNF-α [114]. In choriocarcinoma cells, TNF-α can promote angiogenesis by activating the PIGF/VEGFR1 and VEGFA/VEGFR2 pathways [115]. Recent studies have suggested that TNF-α can exert both anti-angiogenic and pro-angiogenic effects in different tumor tissues, likely due to differences in its expression.

#### Interleukins in the tumor microenvironment play an important role in promoting tumor angiogenesis

Interleukins (ILs) are a class of cytokines that play an important role in the maturation, activation, proliferation, and regulation of immune cells. In addition, they participate in various physiological and pathological processes. IL-1 is an inflammatory cytokine that is important for tumor angiogenesis. IL-1 was originally named hemopoietin-1 because of its angiogenic effect [116]. The IL-1 family cytokines bind to their receptors and activate downstream signaling pathways. Upon activation, MyD88 forms a complex with interleukin-receptor associated kinase 4 to activate downstream MAPK and IKK/NF-κb signaling pathways [117]. The secretion of IL-1α by colorectal cancer cells can increase the proliferation and tube formation capacity of HUVECs [118]. Furthermore, IL-1α exerts pro-angiogenic effects in glioma, pancreatic cancer, and prostate cancer cells by activating JNK signaling and increasing VEGF expression [119–122]. As IL-1β and IL-1 bind to the same receptor, both can promote angiogenesis by inducing the expression of ANG-1, Tie-2, and VEGF via JNK and p38 MAPK signaling [123]. In melanoma cells, both IL-1α and IL-1β can promote tumor angiogenesis by activating NF-κB signaling pathways to induce the expression of IL-6, IL-8, intercellular adhesion molecule-1, and tissue factor [124]. Thus, IL-1 signaling promotes angiogenesis by activating JNK or p38 MAPK and NF-κB signaling, and the IL-1 receptor antagonist inhibits tumor angiogenesis by blocking IL-1 signaling [125]. In addition, several other members of the IL-1 family participate in tumor angiogenesis. IL-33 promotes colorectal cancer cell growth and liver metastasis by regulating the tumor microenvironment [126]. IL-33 can also activate endothelial cells, increase vascular permeability, and promote angiogenesis via ST2/TRAF6-Akt-eNOS signaling. Furthermore, IL-33 can phosphorylate VE-cadherin to facilitate disruption of intercellular junctions of endothelial cells and enhance vascular permeability [127]. Lastly, IL-33 can downregulate the expression of tight junction proteins such as occludins, and reduce the barrier integrity of endothelial cells [128]. In glioma cells, IL-18 facilitates VEGF-induced migration and forms a positive feedback loop wherein VEGF can upregulate IL-18 expression via ERK1/2 signaling [129]. IL-18 can promote angiogenesis via Src and JNK signaling pathways [130]. However, a few studies have demonstrated that IL-33 and IL-18 can exert anti-angiogenic effects in different tissues according to the local environment. Recent studies have showed that IL-36γ can enhance the tube formation capacity of HUVECs in a VEGF-dependent manner [131]. TGF-β can increase the ability of IL-37 to bind to the activated protein receptor-like kinase 1 receptor complex, and upregulates the expression of angiogenesis-related genes [132]. In addition, IL-37 can induce proliferation and migration of endothelial cells, increase capillary formation, and promote the survival of endothelial cells via ERK1/2 and AKT signaling [133]. IL-6 promotes angiogenesis via IL-6/STAT3/VEGFA signaling in hepatocellular carcinoma, cervical cancer, and glioma carcinoma cells [134–136]. IL-8 can increase endothelial cell migration via PI3K/Rac1/RhoA signaling, and promote angiogenesis in prostate cancer cells by increasing MMP9 expression [137, 138]. Furthermore, IL-8 can be used as an independent prognostic factor for patients with early-stage prostate cancer [139]. Lastly, IL-8 can promote tumor angiogenesis in non-small-cell lung cancer, colorectal cancer, and glioma cells [140–142]. IL-17 can promote tumor angiogenesis [143]. It can increase VEGF expression via activation of STAT3 signaling in non-small-cell lung cancer and glioma cells, and IL-6, IL-8, and VEGF expression via activation of STAT1 signaling in lung adenocarcinoma cells [144–146]. Moreover, IL-17 can stimulate fatty acid β-oxidation in endothelial cells [147]. A few studies have also demonstrated that IL-22 possess pro-angiogenic activity [148]. In conclusion, ILs found in the tumor microenvironment can promote angiogenesis.

### Non-coding RNA

**Fig. 5:**
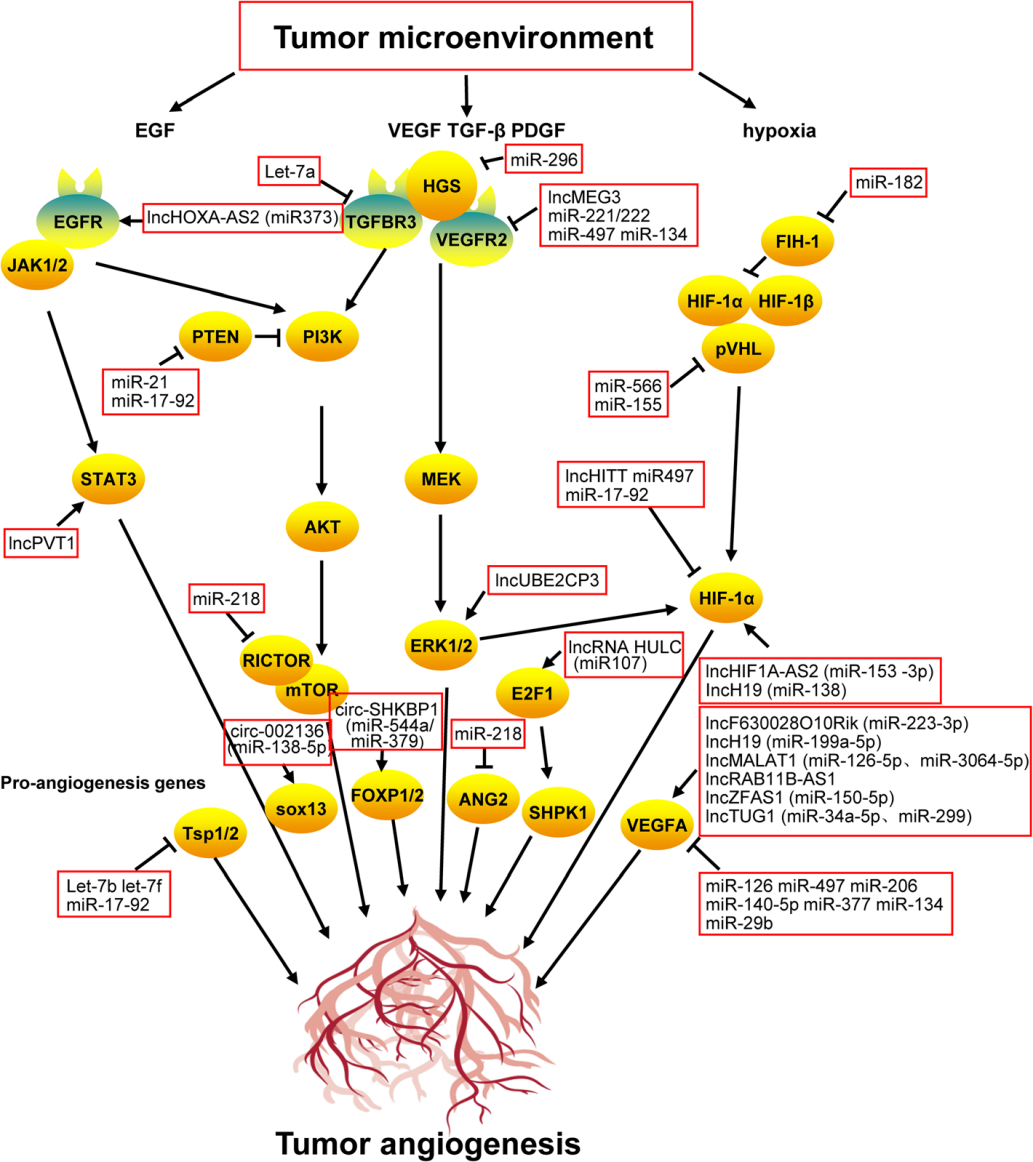
Role of non-coding RNA in regulating tumor angiogenesis

Tumor angiogenesis is not only regulated by angiogenic factors and cytokines in the tumor microenvironment, but also through various intracellular components such as non-coding RNAs. These molecules can enter tumor cells via exosomal or non-exosomal transport mechanisms [149, 150]. The role of non-coding RNAs in the development and progression of tumors has been extensively reported [151–153]. In addition to tumor cell growth, invasion, metastasis, metabolism, and immune escape, non-coding RNAs play an important role in tumor angiogenesis (Fig. 5).

Long non-coding RNA (lncRNA) is an endogenous RNA molecule with a > 200 nt in length, without protein-coding capacity [154]. The number of lncRNAs in the human genome is higher than that of protein-coding genes or small molecule RNAs (such as microRNAs or miRNAs) [155]. Several studies have demonstrated that lncRNAs can regulate tumor angiogenesis. In lung cancer cells, lncRNA F630028O10Rik reduces angiogenesis by inhibiting VEGFA secretion and tumor growth. This activity is similar to that of miR-223-3p [156]. LncRNA UBE2CP3 promotes angiogenesis in hepatocellular carcinoma cells by activating ERK/HIF-1α/VEGFA signaling [157]. LncRNA H19 binds to miR-138 through the mechanism of competing endogenous RNA (ceRNA), facilitating *HIF-1α* RNA stability and VEGFA expression to promote angiogenesis [158]. LncRNA H19 also interacts with miR199a-5p to increase *VEGFA* mRNA expression and promote angiogenesis [159]. In contrast, lncRNA PVT1 upregulates VEGFA expression by binding to phosphorylated STAT3 and stabilizing p-STAT3 protein expression [160]. LncRNA HOXA-AS2 promotes vasculogenic mimicry in glioma cells by binding to miR-373 and increasing the expression of EGFR and its downstream effectors VE-cadherin, MMP2, and MMP9 [161]. In colorectal cancer cells, lncRNA MALAT1 interacts with miR-126-5p in a ceRNA-depended mechanism to induce VEGFA expression and promote angiogenesis. Additionally, lncRNA MALAT1 can reverse the inhibitory effect of miR-3064-5p on VEGFA in a ceRNA-dependent manner [162, 163]. In gastric cancer cells, lncRNA MALAT1 can promote angiogenesis and vasculogenic mimicry via VE-cadherin/β-catenin signaling [164]. LncRNA ZFAS1 promotes angiogenesis via miR-150-5p/VEGFA signaling [165]. In breast cancer cells, lncRNA RAB11B-AS1 recruits RNA polymerase II to upregulate VEGFA and ANGPTL4 expression and promotes tumor angiogenesis [166]. In hepatocellular carcinoma cells, lncRNA HULC promotes angiogenesis via miR-107/E2F1/SPHK1 signaling [167]. In nasopharyngeal carcinoma, lncRNA HOTAIR promotes angiogenesis via GRP78-mediated upregulation of VEGFA and ANG2 expression [168]. LncRNA TUG1 can also promote tumor angiogenesis; however, its mechanism is different in different tumor cells. LncRNA TUG1 upregulates VEGFA expression by binding to miR-34a-5p and miR-299 in in hepatocellular carcinoma cells and glioma cells, respectively [169, 170]. LncRNA MEG3 can modulate VEGFR2 expression and alter the biological activity of endothelial cells. In breast cancer cells, lncRNA MEG3 suppresses proliferation and angiogenesis by inhibiting AKT signaling [171, 172]. In endothelial cells, lncRNA HIF1A-AS2 binds to miR-153-3p to increase HIF-1α expression and promote angiogenesis. In contrast, lncRNA HITT inhibits angiogenesis in colorectal cancer cells by inducing the cleavage of YB-1 from the 5′-UTR region of HIF-1α protein, resulting in HIF-1α protein degradation [173, 174]. Recent studies have showed that most lncRNAs modulate tumor angiogenesis by binding to miRNA and regulating the expression of angiogenesis-related genes. The recent discovery of the ability of lncRNA to bind proteins and secrete small peptides to regulate physiological functions has paved the way to understand the mechanism of lncRNA-induced tumor angiogenesis.

miRNAs are single-stranded small non-coding RNA molecules of approximately 22 nt in length. Mature miRNAs are processed from single-stranded RNA precursors (70–90 nt) with a hairpin structure by the Dicer enzyme. Recent studies have demonstrated that miRNAs can regulate multiple genes. A mature miRNA can regulate the expression of its target genes either by degrading the target mRNA or by inhibiting protein translation [175–177]. The role of miRNAs in tumor angiogenesis has also been extensively reported in recent years. miR-155 downregulates *VHL* expression, stabilizes HIF-1α protein expression, and promotes angiogenesis in triple-negative breast cancer and renal cancer cells [178, 179]. miR-566 downregulates *VHL* expression and upregulates HIF-1α and VEGF expression to promote angiogenesis in glioma cells [180]. miR-21 degrades PTEN mRNA and upregulates HIF-1α and VEGF expression via AKT and ERK1/2 signaling. miR-182 target PHDs and FIH1 to promote angiogenesis in prostate cancer cells [181, 182]. miR-296 inhibits the degradation of VEGFR2 and PDGFRβ by targeting HGS and promotes tube formation of endothelial cells [183]. The miR-17-92 cluster targets Tsp1, PTEN, and HIF-1α to promote angiogenesis in lung cancer cells [184, 185]. In prostate cancer cells, let7b and let7f can promote angiogenesis by targeting TIMP-1 and Tsp1/2 [186]. The angiogenic activity of miR-221/222 is particularly interesting. miR-221/222 promotes angiogenesis by targeting TIMP2 and upregulating MMP expression. Alternatively, it directly targets VEGFR2 and inhibits angiogenesis in renal cancer cells [187, 188]. In breast cancer cells, miR-126 targets VEGFA and PIK3R2 to reduce VEGF/PI3K/AKT signaling and inhibit tumor angiogenesis [189]. miR-497 targets multiple genes, including *VEGFR2*, *VEGFA*, *AEG-1*, and *HIF-1α* to inhibit tumor angiogenesis [190–192]. miRNA let-7a inhibits TGF-β signaling and tumor angiogenesis by targeting TGFBR3. miR-328 targets CD44 and inhibits endothelial cell activity and tube formation ability of breast cancer cells. miR-135a inhibits tumor invasion, metastasis, and angiogenesis in glioma cells by targeting FAK and inhibiting VEGF signaling. miR-29b targets VEGF, MAPK/ERK, and PI3K/AKT signaling pathways to inhibit angiogenesis in endometrial cancer cells. miR-206 inhibits angiogenesis by targeting VEGF and MAPK3 in triple-negative breast cancer cells. miR-140-5p directly targets VEGFA and suppresses VEGFA/MMP2 signaling to inhibit angiogenesis in colorectal cancer and glioma cells [186, 193]. miR-377 targets VEGF and CD133, and inhibits tumor growth and angiogenesis in esophageal cancer cells [194]. miR-134 targets the 3′-UTR of VEGFA and VEGFR1 and suppresses VEGFA/VEGFR1/AKT signaling to inhibit angiogenesis in osteosarcoma cells [195]. miR-218 downregulates the RICTOR/mTOR/HIF-1/VEGF signaling pathway by degrading RICTOR mRNA. In addition, miR-218 inhibits angiogenesis in glioma cells by targeting ANG2 and ROBO1 [196–198]. Owing to the large number of miRNAs and the ability of a single miRNA molecule to recognize multiple target genes, it is speculated that several other miRNAs will be found to be associated with tumor angiogenesis in the future.

In addition to lncRNAs and miRNAs, circRNAs have gained immense attention in recent years [199, 200]. Numerous studies have demonstrated that circRNAs play an important regulatory role in tumor angiogenesis, in addition to its role in tumor growth, invasion, and metastasis. In glioma cells, circ-002136 binds to miR-138-5p to rescue SOX13 inhibition and promote angiogenesis [201]. The RNA-binding protein MOV10 binds to circ-DICER1 and enhances its association with miR-103a-3p/miR-382-5p, leading to increased expression of its downstream target, *ZIC4*, promoting glioma tumor growth, migration, and angiogenesis [202]. circ-SHKBP1 forms RNA-induced silencing complexes with miR-544a and miR-379 to rescue inhibition of downstream effectors FOXP1 and FOXP2 and promote angiogenesis in glioma cells [203]. In hepatocellular carcinoma cells, circ-100,338 promotes endothelial cell permeability and tube formation; however, the precise regulatory mechanism is unknown [204]. We believe that more circRNAs with regulatory effects on tumor angiogenesis will be discovered in future.

### Clinical medication for tumor angiogenesis

Angiogenesis is an integral part of tumor progression and plays a pivotal role in tumor growth and metastasis. In the 1970s, Professor Folkman proposed that tumor growth and metastasis rely on angiogenesis, and inhibition of angiogenesis can be used as a therapeutic strategy for tumor treatment [205]. Recently, targeting of pro-angiogenic genes has become a research hotspot for tumor therapy and prevention of tumor expansion. The current FDA-approved anti-angiogenic drugs are categorized into two types based on the number of targets: single-target inhibitors and multi-target inhibitors. VEGF is an important target molecule for antitumor angiogenesis. In recent years, several monotherapeutic drugs have been used against VEGF (Table 1). Bevacizumab—the first FDA-approved anti-angiogenic inhibitor—is a recombinant humanized monoclonal antibody that is marketed as Avastin, and was developed by Genentech. It was approved by the FDA in February 2004. It can reduce the binding of VEGF to VEGFR and inhibit the growth of blood vessels. It was first approved for the clinical treatment of metastatic colorectal cancer and subsequently approved for that of non-squamous small-cell lung cancer, cervical cancer, ovarian cancer, metastatic breast cancer, and malignant glioma. Ramucirumab is a human IgG1 monoclonal antibody that prevents the proliferation and migration of vascular endothelial cells by inhibiting ligand-induced activation of VEGFR2. Ramucirumab was approved by the FDA in 2014 for the treatment of advanced gastric or gastroesophageal adenocarcinoma, non-small-cell lung cancer, and metastatic urinary tract epithelial cancer [206]. Ziv-aflibercept is a recombinant fusion protein consisting of the VEGF-binding site of VEGFR and the Fc region of IgG1. This drug was manufactured by Sanofi and is used to target VEGFA/VEGFB/PIGF signaling. Ziv-aflibercept was approved by the FDA in August 2012 for use in combination with 5-fluorouracil, calcium folate, and irinotecan for the treatment of metastatic colorectal cancer [207]. Several inhibitors targeting multiple tyrosine kinases have been approved. Axitinib, manufactured by Pfizer, was approved by the FDA in January 2012 for the treatment of advanced renal cell carcinoma [208]. Sorafenib, developed and manufactured by Bayer, was approved by the FDA in December 2005 for the treatment of renal cell and hepatocellular carcinoma and thyroid cancer [209]. Sunitinib is a small-molecule multitarget receptor tyrosine kinase inhibitor developed and manufactured by Pfizer. It was approved by the FDA in 2006 for the treatment of gastrointestinal stromal tumors, advanced renal cancer and metastatic well-differentiated advanced pancreatic neuroendocrine tumors [210]. Regorafenib is a multikinase small molecule inhibitor developed and manufactured by Bayer. It was initially approved by the FDA in September 2012 for the treatment of metastatic colorectal cancer and subsequently approved for that of gastrointestinal mesenchymal tumors and liver cancer. Nintedanib was developed by Boehringer Ingelheim and approved by the FDA in October 2014 for the treatment of idiopathic pulmonary fibrosis and non-small-cell lung cancer [211]. In 2012, cabozantinib was first approved by the FDA for progressive, metastatic thyroid cancer and non-small-cell lung cancer with c-Met amplification. In April 2016, Exelixis announced FDA approval of cabozantinib for the treatment of patients with advanced kidney cancer. Pazopanib was developed by GlaxoSmithKline and initially approved by the FDA in October 2009 for the treatment of advanced renal cancer and subsequently approved for that of advanced soft tissue sarcoma, epithelial ovarian cancer, and non-small-cell lung cancer [212]. Several drugs targeting angiogenesis are currently undergoing clinical trials.

Although anti-angiogenic drugs have proven to be effective in inhibiting tumor progression, a single anti-vascular treatment strategy cannot eliminate the tumor. Firstly, the regulatory network of angiogenesis is complex. Therefore, inhibition of a single signaling pathway may be compensated by other potential angiogenic mechanisms. Several studies have demonstrated that VEGF-C and VEGF-D can promote angiogenesis and tumor progression even when VEGFA activity is suppressed. Moreover, clinical data have revealed that despite receiving anti-VEGF treatment with bevacizumab, the plasma levels of FGF and PDGF in patients were increased. These factors can promote tumor angiogenesis. Furthermore, the side effects of bevacizumab administration include proteinuria, hypertension, and bleeding from the perforated gastrointestinal tract. Post-treatment examination of patients revealed increased drug resistance and tumor metastasis [213]. The side effects of sunitinib include lung toxicity, difficulty in breathing, and coughing, and those of pazopanib include cardiovascular toxicity, hypertension, and abnormal ventricular polarization[1]. In addition, patients consuming anti-angiogenic drugs can develop drug resistance. However, because anti-angiogenic drug resistance is not caused by genetic factors, it can be reversed. The mechanisms of drug resistance include angiogenesis, tumor vascular protection, increased aggressiveness of tumor cells, and increased tumor metastasis through different angiogenesis patterns [214]. Increased expression of angiogenic genes, increased secretion of various angiogenic factors, and increased recruitment of cells derived from angiogenic bone marrow can develop anti-angiogenic resistance [215]. Therefore, more attention is required to address issues such as drug resistance and side effects of anti-angiogenic drugs.

## Discussion and future directions

This paper reviews factors that influence angiogenesis in the tumor microenvironment. The tumor microenvironment consists of numerous pro-angiogenic factors, including VEGF, bFGF, and PDGF. These factors are secreted by tumor cells or tumor-infiltrating lymphocytes or macrophages, and can activate pro-angiogenic signaling pathways to promote tumor angiogenesis, growth, invasion, and metastasis. In addition, inflammatory cytokines in the tumor microenvironment play an important role in promoting tumor angiogenesis. Previous studies have showed that IFNs, TNF, and TGF-β can exert antitumor effects. However, a few studies have demonstrated that these factors are capable of promoting angiogenesis and tumor progression. These results indicate the diverse role of cytokines in tumorigenesis and development. Several members of the IL-1 family promote tumor angiogenesis. IL-1 signaling promotes angiogenesis by upregulating VEGF and angiogenesis-related molecules through the activation of JNK or p38 MAPK and NF-κB signaling. IL-6, IL-8, and IL-22 can also promote tumor angiogenesis by regulating the expression of angiogenic factors. Furthermore, a hypoxic microenvironment can promote tumor growth, invasion, metastasis, immune escape, and angiogenesis. Therefore, co-targeting of hypoxic factors and anti-angiogenic factors can improve tumor outcomes. In a study on glioma xenografts, the researchers found that co-treatment with HIF-1α inhibitors and bevacizumab showed a higher antitumor effect than treatment with bevacizumab alone [216]. HIF-1α is an upstream regulator of several angiogenic factors and can directly induce transcription of angiogenic factors to promote tumor angiogenesis. Additionally, multiple hypoxia-induced ncRNAs can promote tumor angiogenesis by regulating the expression of angiogenic factors. The tumor microenvironment is replete with angiogenic factors. Therefore, treatment of cancer cells with drugs that target multiple angiogenic factors may yield better results. Therapeutic strategies to inhibit the secretion of these angiogenic factors can be useful to treat tumors. In addition to cytokines, inflammatory factors can promote tumor angiogenesis. Therefore, reducing inflammation in the tumor microenvironment or decreasing the secretion of certain inflammatory cytokines can produce an anti-angiogenic effect. Current understanding of the tumor microenvironment is limited. The detailed regulatory mechanisms of tumor angiogenesis by cytokines and hypoxia in the tumor environment are not well understood. Therefore, an in-depth investigation of the role of inflammatory cytokines in the tumor microenvironment may provide new therapeutic strategies for the treatment of tumor angiogenesis.

## Data Availability

Not applicable.

## Abbreviations

TNF: Tumor necrosis factor
IFN: Interferon
TGF: Transforming growthfactor
Th1: T helper type 1
ILs: Interleukins
MMPs: Metalloproteinases
VEGF: Vascular endothelialgrowth factor
FGF: Fibroblast growth factor
FGFR: Fibroblast growth factor receptor
PDGF: Platelet-derived growth factor
BMPs: Bone morphogenetic proteins
PIGF: Placental growth factor
CXCL-12: C-X-C motif chemokine 12
ECM: Extracellular matrix
EMT: Epithelial-msenchymal transition
FDA: Food and Drug Administration
HIF: Hypoxia-induced factor
PHD: Proline hydroxylase
FIH: Factor-inhibiting HIF
VHL: Hippel-Lindau
HREs: Hypoxic response elements
EBV: Epstein-Barr virus
HUVEC: Human umbilical vein endothelial cell
lncRNA: Long non-coding RNA
miRNA: MicroRNA
circRNA: Circular RNA
